# Ageing-Associated Oxidative Stress and Inflammation Are Alleviated by Products from Grapes

**DOI:** 10.1155/2016/6236309

**Published:** 2016-02-29

**Authors:** K. S. Petersen, C. Smith

**Affiliations:** Department of Physiological Sciences, Stellenbosch University, Stellenbosch 7600, South Africa

## Abstract

Advanced age is associated with increased incidence of a variety of chronic disease states which share oxidative stress and inflammation as causative role players. Furthermore, data point to a role for both cumulative oxidative stress and low grade inflammation in the normal ageing process, independently of disease. Therefore, arguably the best route with which to address premature ageing, as well as age-associated diseases such as diabetes, cardiovascular disease, and dementia, is preventative medicine aimed at modulation of these two responses, which are intricately interlinked. In this review, we provide a detailed account of the literature on the communication of these systems in the context of ageing, but with inclusion of relevant data obtained in other models. In doing so, we attempted to more clearly elucidate or identify the most probable cellular or molecular targets for preventative intervention. In addition, given the absence of a clear pharmaceutical solution in this context, together with the ever-increasing consumer bias for natural medicine, we provide an overview of the literature on grape (*Vitis vinifera*) derived products, for which beneficial effects are consistently reported in the context of both oxidative stress and inflammation.

## 1. Introduction

With ageing, the capacity of the body to function optimally declines. There is a combination of genetic and lifestyle factors which may either accelerate or slow down the ageing process. A number of chronic diseases are associated with advanced age: these include cardiovascular disease (CVD), diabetes, metabolic syndrome, and Alzheimer's disease. This results in an exponential increase in the disease burden on modern society, relative to a few decades ago, due to longer life expectancy. The World Health Organisation states that from 2015 to 2050, the proportion of the world's population over 60 years will increase from 12% to 22% [1]. Taking into consideration that predominant research in the field of age-associated diseases considers the sixth decade of life to be a risk factor for the rapid progression and onset of age-associated diseases, the disease burden will almost double in the next 35 years. It is thus vital not only to elucidate the causes and progression of these chronic conditions, but also to actively search and investigate potential preventative therapies that may slow the processes contributing to physiological ageing.

Although the ageing-associated chronic disease states are each uniquely complex in terms of their aetiology, development, and progression, they do share common aetiologies which stem from two main entities, namely, cumulative oxidative stress and chronic inflammation. Briefly, oxidants are produced by normal cell metabolism and various physiological responses. However, when the production of oxidants outweighs the capacity of endogenous antioxidant systems, oxidative stress is incurred. Furthermore, while inflammation is crucial for repair of tissue injury and primary defence against invading pathogens and chemicals, it also results in unintended detriment to previously uninjured cells. Although these are necessary systems in the body, both oxidative stress and the inflammatory response, if unchecked, can have detrimental consequences which have been linked to accelerated ageing and the progression of age-associated disease. We postulated that the effects of the inflammatory immune system and oxidative stress on allostatic load are interlinked. This has led us to investigate the potential of antioxidants as treatment options to attenuate the cumulative effects of both oxidative stress and chronic low grade inflammation. Given the modern consumer bias for natural medicines, we focused on a group of plant medicines which are consistently associated with beneficial effects on these processes in the literature, grape-derived polyphenols. In this review, we will provide a more in-depth review of interconnected molecular mechanisms of oxidative stress and inflammation in the physiological ageing process, before moving our focus to a discussion of the merits of these plant medicines as potential preventative therapy in this context.

## 2. Contribution of Oxidative Stress to Premature Ageing

In the context of ageing, reactive oxygen and nitrogen species (RONS) are generally the major molecules which contribute significantly to oxidative stress. The most frequently studied free radicals are superoxide (O_2_^−^), hydrogen peroxide (H_2_O_2_), hydroxyl radical (^∙^OH), peroxynitrite (ONO_2_^−^), and nitric oxide (NO). The generation of these free radicals is necessary as they are essential for host defence: phagocytic cells use ROS to digest invading pathogens and debris. Furthermore, they act as signalling molecules, regulating cell growth and apoptosis, adhesion, and differentiation. More specifically, RONS are formed during processes such as the mitochondrial electron transport chain as well as enzyme systems such as cytochrome P450, lipoxygenase and cyclooxygenase, the NADPH-oxidase complex, xanthine oxidase, and peroxisomes [2]. In contrast to these roles in growth, repair, and immune functions which are all beneficial to the host, these molecules also have the ability to oxidise signalling molecules, DNA, macromolecules, and cell structures such as lipid membranes of healthy host cells, all of which are to the detriment of these cells. Usually, each cell has defence mechanisms to counteract the occurrence of oxidative stress. These endogenous enzymatic antioxidant defences include superoxide dismutase (SOD), glutathione peroxidase, catalases, glutathione/TrxR, and peroxiredoxins. An appropriately nutritious diet is vital to maintain these systems, as these natural antioxidants are supplemented or replenished by antioxidant constituents of various fruits and vegetables. It is only when the capacity of the body's antioxidant defences is outweighed by the rate of production of free radicals that oxidative stress is incurred by unquenched free radicals which can alter surrounding cell structures and environment.

With advancing age, various factors, among these is a natural progressive decline in endogenous antioxidant capacity, cause disruption in the balance between pro- and antioxidant mechanisms and RONS accumulate beyond the normal endogenous antioxidant system's quenching capacity, resulting in cumulative oxidative stress. Eventually this causes cell damage which cannot be repaired by internal mechanisms, leading to loss of organ mass and functionality, which ultimately culminates in system dysfunction [3]. Indeed, long-term oxidative stress states have been linked to various diseases associated with advanced age, most notably diabetes, chronic obstructive pulmonary disease (COPD), cardiovascular disease, cancer, diabetes, and asthma [4]. Many of these diseases are not only associated with ageing anymore, but also with the high-obesity, high-stress, and sedentary modern lifestyle (albeit perhaps in milder form) [5]. It is thus vital to address the prevention of these detrimental long-term outcomes, as they are afflicting not only the aged, but also relatively young populations, such as university students [6, 7].

The aetiological mechanism(s) of the various chronic diseases mentioned are each different, as each disease has its own complexities. However, chronic cumulative oxidative stress is a common factor in these diseases, highlighting this system as a vital therapeutic and/or preventative medicine target [8].

Even in the absence of age-associated disease, various theories have linked oxidative stress directly to the normal ageing process. We briefly mention two here. Firstly, the Free Radial Theory of Ageing proposes that the presence of free radicals and their effects on cells are one of the causes of cell ageing and subsequent cell death, which in turn lead to loss of organ mass and other features of whole organism ageing. This theory was first suggested by Harman in 1956 [9], who hypothesized that irradiation of cellular components resulting from the liberation of ^∙^OH and ^∙^HO_2_ radicals would lead to dysfunction and mutations which cannot be reversed and thus result in ageing. This theory was later supported and expanded as research began elucidating the details of his proposed theory, for example, by describing the role of SOD [10]. Phenomenally, a very recent meta-analysis [11] confirmed the sustained validity of this theory, 60 years later. Of course, given the technological advances made over this period, it is no surprise that subsequent research has added more detail on mechanisms in support of this theory, although many unresolved questions remain [12].

Secondly, the Replicative Senescence Theory is based on the hypothesis that oxidative stress induces cell death and/or senescence, which necessitates an increase in the rate of cell replication. This in turn accelerates the detrimental effects associated with repeated cycles of mitosis. Telomere length and its accelerated shortening due to reparative replication is the basis of this theory, which was first described by Hayflick in 1965, in response to the observation of a decline in functionality of cell cultures (fibroblasts) which had undergone numerous cell divisions [13]. This phenomenon was subsequently confirmed by experiments on primary peripheral blood mononuclear cells (PBMCs) and fibroblasts from a largely aged population with increased risk for vascular dementia [14]. In this report, decreased telomere length in both fibroblasts and PBMCs was correlated to risk for dementia induced by stroke. In addition, telomere shortening rate was reported to decrease with increasing antioxidant capacity in fibroblasts of the same population. This theory, together with the subsequent data, presents evidence that, firstly, oxidative stress is responsible for the accelerated shortening of telomeres brought about by more frequent reparative replication and, secondly, that an increase in antioxidant defence capacity could slow down the ageing process. Note at this point that we do not infer that telomere shortening results only from oxidative stress: other mechanisms such as chromosomal instability have indeed been linked to both ageing and pathological conditions such as cancer [15]. However, to remain focused on the main topic of this review, we have limited this discussion to* accelerated* telomere shortening in the context of cumulative oxidative stress.

Holistic interpretation of the two theories introduced above implicates oxidative stress as major causative role player in the damage to cell constituents by oxidising membranes, molecules, and DNA. This initiates a cascade of events leading to the need for either cell growth and replication for repair, or death. This implicates ROS as rate determining factor of cell lifespan due to the direct damage it inflicts. Furthermore, oxidative stress is implicated in causing irreversible damage to the mechanism of replication through accelerated telomere shortening and thus ultimately decreases the capacity of the cell to replicate optimally, or at all. With both repair and replication affected, cell senescence is encouraged.

In keeping with the idea of a holistic approach, consideration of oxidative stress in isolation is insufficient. A basic but practical example of the interplay between the oxidative stress system and another system implicated in ageing, inflammation [16], is data available on detrimental effects of cigarette smoking. In this context, both acute smoking and long-term smoking were shown to overwhelm the glutathione antioxidant defence system within the lungs, which was associated with significant infiltration of inflammatory immune cells in the lungs [17]. This study clearly shows system interaction. Furthermore, in the same study, the severity of this maladaptation increased with duration of habitual smoking (in years) and was exacerbated by natural decreases in antioxidant capacity as experienced with ageing, resulting in increased oxidative stress, illustrating the significance of cumulative damage.

However, before considering these interactions in more detail, the literature providing proof of a role for chronic inflammation in the process of ageing will be briefly reviewed.

## 3. Chronic Low Grade Inflammation Facilitates Premature Ageing

As is the case for oxidative stress, the inflammatory response is a system essential for normal body function [18]. As component of the innate immune response, inflammation is a major first-line defence against infection and injury [18]. Apart from this largely independent, nonspecific role in immunity, inflammation is also vital for many specific immune responses to run its course [19]. However, in the process of repair and restoration after insult, the inflammatory response inadvertently disrupts cellular homeostasis of previously unharmed or unaffected cells [20, 21]. The injury-repair cycle which inflammation regulates is an efficient system during youth, when optimal sensitivity and response to signalling molecules (such as cytokines, growth factors, prostaglandins, and peptides) maintain the general health of circulating immune cells and the tissue microenvironment, with minimum secondary damage. However, during natural chronological ageing, long-term, repeated stimulus-response cycles change the receptor expression levels and thus sensitivity to these molecular stimuli [22]. This may necessitate relatively increased concentrations of any particular stimulus to maintain the required effect. Commonly reported characteristics of the natural ageing process include inflammation or oxidative stress-associated symptoms such as directionally inaccurate chemotaxis, premature or suboptimal respiratory burst, and increased proinflammatory signalling from immune cells, all of which form the basis of immunosenescence [23].

Immunosenescence is the term used to describe ageing of immune cells and the functioning of the immune system as a whole. This occurs naturally with advancing chronological age or as result of lifestyle factors, as already mentioned. There is more than one way in which the immune system is compromised upon ageing. Firstly, immunocompetent cells are derived from hematopoietic stem cells. With ageing, a natural bias develops for stem cells to commit to expansion of the myeloid lineage at the cost of the lymphoid lineage [24]. This results in a shift in the balance of immune cells available to enter the circulation. Secondly, the chronic low grade inflammation associated with ageing recruits larger numbers of cells into circulation from the hematopoietic tissue. However, despite the higher circulating cell counts, phagocyte Toll-like receptor expression and phagocytic capacity are decreased in the aged [22], leaving the immune system with a lower capacity for becoming activated and for responding to a more acute insult, such as viral infection. These maladaptations, which together further predispose the individual to proinflammatory responses, are postulated to stem at least in part from alterations in HPA-axis signalling.

The process of immunosenescence may be accelerated by unhealthy lifestyle, such as psychological stress and obesity. As mentioned before, lifestyle-associated diseases share clinical symptoms associated with normal ageing. Indeed rutin, a potent antioxidant, has been shown to protect against ageing-related metabolic dysfunction [25]. Thus, studies focused on those conditions may provide much insight in terms of ageing and the supplementation options to limit its progress.

For example, in the context of obesity and/or inactivity, cytokines release from adipose tissue macrophages has been demonstrated by many researchers [26–29]. Tumour necrosis factor-*α* (TNF-*α*), interleukin- (IL-) 1*β*, and IL-6 are among some of the cytokines shown to be released from resident macrophages in adipose tissue, resulting in a proinflammatory microenvironment [30]. Furthermore, chronic stress, and in particular psychological stress, is a generally accepted cause of chronic low grade inflammation. For example, in 38 medical students, psychological stress was associated with increased proinflammatory cytokine (TNF-*α*, IL-6, IL-1Ra, and IFN-*γ*) levels, as well as decreased anti-inflammatory regulators (IL-10 and IL-4) [31]. Interestingly, these effects were exacerbated by high anxiety proneness as trait, again suggesting that a cumulative stimulus (in this case lifelong anxiety) further exacerbates the undesirable adaptation.

Also in posttraumatic stress disorder (as extreme form of chronic psychological stress), an initial glucocorticoid hyperresponse is followed by glucocorticoid hyporesponsiveness, which is associated by a relatively proinflammatory state. In this condition of continuous proinflammatory signalling, the feedback systems, which usually downregulate inflammation, adjust overtime and result in maladaptations such as chronic but low grade upregulation of proinflammatory mediators (e.g., IL-6, TNF-*α*, IL-1*β*, and prostaglandin E_2_) [32]. Incidentally, the secretion of the first three is mediated by the NF-*κ*B pathway, which is activated in response to cellular stress [33].

In addition to increased proinflammatory signalling, other more mechanical cellular mechanisms also seem to be compromised over time. For example, inappropriate and/or insufficient neutrophil responses result from its decreased phagocytic capacity, increased basal levels of intracellular calcium, and the resulting reduction in capacity for chemotaxis [34]. The result is a chronic low grade inflammatory status in relative absence of a specific threat which can persist for extended periods of time and cause harm and inefficiency of the system. Thus, although varied in specific causative mechanism, the outcome of all of these suboptimal life events or lifestyle habits, for example, chronic stress leading to glucocorticoid resistance and/or cardiovascular disease and high-calorie diets and inactivity (obesity) leading to insulin resistance and diabetes, is that of a chronic inflammatory state [16].

Of particular interest in the context of ageing is the fact that, apart from the now notorious low grade inflammation as primary culprit, this maladaptation results in a compromised capacity to mount an efficient inflammatory response to acute insults. As overviewed in the review by Weinberger and colleagues [35], several reports from clinical literature support the notion that, in the aged individual, that there is a significant increase in the convalescence period required for recovery from injury and pathogen clearance, as well as a decrease in the quality of repair, thus favouring disease progression and morbidity due to injury. Very recently, Baëhl and colleagues (2015) demonstrated, in a longitudinal study of elderly patients, that the acute stress of a hip fracture had a negative effect on neutrophil function immediately after injury. While some neutrophil functions (chemotaxis, phagocytosis) were recovered over time, several others (superoxide production, complement C5A and CD11b receptor level, and cytokine secretory profile) were still impaired even 6 months after injury. From this, the authors concluded that the acute stress had a long-term negative effect on neutrophil responses, which negatively influenced clinical outcomes, such as the resolution of long-term inflammation, recovery, and susceptibility to opportunistic infections [36].

It is thus clear that ageing is an inflammatory-mediated process. However, from the inflammation/ageing literature, it is clear that inflammation and oxidative stress cannot be separated as causative factors in this context. For example, the ageing-related shift in balance between the glucocorticoid and inflammatory systems has been linked to increased ROS production, which in turn exacerbates low grade immune activation [37].

## 4. Links between Inflammation and Oxidative Stress in the Ageing Process: Identifying Therapeutic Targets

From the above sections it is evident that, in ageing, oxidative stress and inflammation are interdependent mechanisms. We postulate that unravelling and understanding of these intricate links between the two responses hold the answer to identification of the major contributor(s) to allostatic load and maladaptation associated with ageing and age-related pathology.

Generally, repeated exposure to reactive oxygen and nitrogen species causes cell damage and thus a proinflammatory signalling response. For example, in aged mice, unquenched reactive oxygen and nitrogen species act as proinflammatory signalling molecules and mediators of inflammation within the cell itself [38]. More specifically, oxidative damage to cells prompts the release of TNF-*α* from these damaged cells [21]. Binding of TNF-*α* to cell surface TNF-*α* receptors activates the NF-*κ*B inflammasome, which results in the further production of other proinflammatory cytokines, most notably IL-1*β*. Incidentally, TNF-*α* specifically has also been implicated in ROS-mediated upregulation of adhesion molecules which facilitate the infiltration of immune cells into tissue [39], with more on this later. Upregulation of inflammation via the NF-*κ*B inflammasome is probably the main aetiological mechanism for age-related chronic conditions with an inflammatory component. Indeed, TNF-*α* upregulation, which is a direct result of increased flux through the inflammasome, has been implicated as causative factor in cardiovascular disease [40]. Of particular relevance to the topic of ageing, the NF-*κ*B inflammasome has regulatory roles in cell growth, survival, and proliferation. However, as recently reviewed [41], ROS production may have either inhibitory or stimulatory roles in the NF-*κ*B pathway, suggesting a dose-dependency of the effects of ROS. This unfortunately also means that development of an intervention strategy/product to modulate this target mechanism is no simple feat and will have to be approached in a very tightly controlled “modification range.”

From the literature consulted, it is clear that bidirectional communication is in place. For example, both neutrophils and macrophages are producers of oxidants* via* the NADPH-oxidase system [42]. The NADPH-oxidase (NOX) proteins aid the transport of electrons across biological membranes and are found in all cells [43]. They are also one of the major generators of ROS in all cells. Particularly, these proteins are the predominant ROS producers in phagocytic cells, a process required for the normal respiratory burst that phagocytes use to kill pathogens and digest cell debris.

Furthermore, activated neutrophils release myeloperoxidase (MPO), which contributes to the formation of hypochlorous acid (HOCL) by acting as a catalyst when reacting with hydrogen peroxide (H_2_O_2_). This directly increases the production of ROS [44]. The oxidative burst of neutrophils in itself releases oxidants such as H_2_O_2_, which are harmful to healthy cells and tissue [45]. Besides directly increasing the production of reactive oxygen species itself which causes damage to surrounding cells, MPO specifically has been implicated as a risk factor for coronary artery disease, due to its capacity for oxidation of lipid membranes [46]. The increased oxidant concentration due to immune cell functions, as well as the resultant cell damage, results in increased metabolism in surrounding healthy tissue.

Also at gene level, role players have been elucidated in the context of a ROS-inflammation link. For example, sirtuins are Class III histone deacetylases which are responsible for the deacetylation at N-epsilon lysine residues, a reaction which consumes NAD+. SIRT1 specifically is a sirtuin commonly associated with antioxidant function [47]. Its regulation of oxidative stress is threefold: firstly, it stimulates the expression of antioxidants* via* the fork-head box protein O (FOXO) pathway. Secondly, it is involved in inhibiting the NF-*κ*B signalling pathway. In contrast, however, excessive ROS can inhibit SIRT1 activity by oxidatively modifying its cysteine residues and thereby releasing its inhibition of the NF-*κ*B pathway [47]. It is thus theoretically possible for cumulative stress to downregulate SIRT1 activity in the ageing process. Thirdly, SIRT1 has also been implicated in regulation of apoptosis by deacetylating p53 to inhibit p53-dependent transcription in models of cellular stress [48]. This tripartite role defines SIRT1 as another important molecular target in the context of both normal and accelerated ageing.

Apart from these targets related to cellular signalling, cell functional capacity should also be a focus. A striking example of the oxidative stress, inflammation link in ageing, is the decreased capacity for neutrophil chemokinesis reported in the elderly both in terms of motility and accuracy [49]. Normally, immune cells are attracted by chemotactic signals from injured tissue, to migrate to sites of injury or pathogen invasion. During this chemokinetic response, immune cells, typically neutrophils and classically activated macrophages, migrate through tissue toward the site of injury. This movement is facilitated largely* via* adhesion molecules such as the beta-integrins and I-CAM1 in the case of neutrophils [21]. However, as mentioned earlier, expression of adhesion molecules on neutrophils increases with ageing [39], so that their movement is slowed. In addition, due to yet unclear mechanisms, but most probably due to adaptation of cellular “homing” molecules, directional accuracy of neutrophil migration is also compromised in the elderly. Sapey et al. showed that inaccurate neutrophil migration was causally associated with increased constitutive phosphoinositide 3-kinase (PI3K) signalling [49]. This results not only in inefficient inflammation due to prolonged response time, but the directional inaccuracy of movement also results in mechanical and oxidative damage to relatively more cells in the path of the migrating inflammatory cell [49].

It is clear that both oxidative stress and inflammation are able to induce and exacerbate one another (both indirectly and directly). Furthermore, regardless of the primary signal or which pathway was activated first, these interrelated processes form a vicious cycle which is difficult to target therapeutically because of its complexity. However, it is also this interrelated nature of the two systems that has led us to investigate the possibilities of antioxidants as treatment options to attenuate the cumulative effects of oxidative stress and in turn low grade chronic inflammation.

## 5. Are Grapes the Answer to Prevention of Ageing?

Despite the huge range of nonsteroidal and natural product anti-inflammatories on the market today, the scientific literature shows a conspicuous lack of consistent support for any specific medication. This is perhaps at least in part due to the fact that researches investigating these products cannot keep up with the rate at which new ones are pushed onto the market. Hopefully new legislation on the control of these substances will affect this trend to the benefit of the consumer, by allowing for (or demanding) appropriate testing of these products.

Nevertheless, antioxidants are being used almost routinely by many individuals who wish to supplement for enhancement of general health or as adjuvant therapy in conjunction with more mainstream, pharmaceutical medication. Although they are generally not regarded as a primary therapeutic option, antioxidants may hold particular potential in the realm of preventative medicine. The potential benefits of appropriate antioxidant supplementation are vast, especially when considering the connection between oxidative stress and inflammation. An antioxidant with the capacity to modulate inflammatory status can thus be beneficial to both normal ageing individuals and those suffering from lifestyle-associated diseases.

A comprehensive search of the scientific literature revealed that grape-derived antioxidants are consistently reported to have high benefit and low risk in the context of both oxidative stress and inflammation. These positive results are further strengthened by the fact that these consistent findings were reported across many different models and using a variety of different preparations, ranging from relatively crude extracts to highly purified ones. For example, in terms of purified polyphenols, resveratrol, one of the best-known polyphenols present in grapes as well as other plants, is commonly linked to anti-inflammatory [50], antioxidant [51, 52], and thus by implication antiageing effects in the scientific literature, as well as anecdotally. Indeed, a recent paper [53] elucidated a role for resveratrol to protect against inflammatory damage via SIRT1 inhibition of the NF-*κ*B pathway (a mechanism discussed above in the context of ageing). In addition, more advanced studies have been undertaken on this polyphenol to better understand the relationship between the chemical structure of resveratrol and its biological activity, especially in terms of oxidant scavenging [54]. Also, pharmaceutical groups have been working on optimisation of delivery systems for resveratrol [55]. Such information may further advance the popularity of this very promising natural product with the pharmaceutical industry, to the benefit of consumers. The phenomenal frequency at which new papers on resveratrol appear, all providing evidence of positive effects in this context, suggests that this particular polyphenol should be investigated in the context of ageing as a matter of urgency.

Even more promising than the many positive effects described for resveratrol above is the fact that resveratrol is only one from a range of equally beneficial substances contained in grapes. The flavonoids quercetin and dihydroquercetin (DHQ), as well as proanthocyanidins and anthocyanins, all of which are present in grapes and a variety of other plant sources, have similarly been linked to both antioxidant and anti-inflammatory effects [50, 52]. To date, despite appearance of a few very promising reports in this context, ageing specifically has not been the focus of many studies investigating these substances. Therefore, for the purpose of this review, we provide a comprehensive overview of the few existing ageing-related studies in this context that were available to us. Results from relevant papers that did not have ageing as a focus were also included, where those results contribute to our understanding of the role of grape-derived polyphenols in the oxidative stress, inflammation link in the context of prevention or deceleration of the ageing process.

When considering* in vivo* studies on ageing as a starting point, resveratrol (0.1 *μ*M to 2.5 *μ*M) exhibited a clear dose-dependent effect on longevity in fish with known short lifespan: resveratrol supplemented fish almost doubled their expected 13-week lifespan and continued to produce healthy offspring long after all control fish had died. Even though resveratrol supplementation was only started in adulthood in this study (i.e., it compares to when humans might start to consider supplementation), it effectively delayed age-dependent compromise of locomotor and cognitive performance and reduced expression of neurofibrillary degeneration in the brain [56]. This result of improved neural morphology was recently further substantiated in an aged rat model, where chronic resveratrol treatment prevented detrimental changes in dendritic morphology which is linked not only to ageing, but also to Alzheimer's disease [57]. Similarly, 2 months of ingesting polyphenols in the form of 10% grape juice resulted in enhanced potassium-evoked dopamine release and cognitive performance in aged rats [58].

A more recent review [59] provides more insight into the potential mechanisms by which age-related cognitive disorders may be curbed by grape polyphenols. Some of these mechanisms at first seem unrelated to the scope of this review, for example, preventing of amyloid-beta deposition associated with Alzheimer's dementia [60]. However, recent research suggests a role for inflammation in the development of the disease [61], while natural antioxidants have been linked to prevention of amyloid-beta deposition [62]. Together, these data suggest that even these seemingly unrelated mechanisms may be interconnected to either inflammation, or oxidative stress, or both. However, more clearly in context of this review, resveratrol was reported to increase NO production, resulting in vasodilation [59, 63], which may play a role in the maintenance of central circulation and thus perhaps slower degenerative central processes, as has indeed been reported for resveratrol, as mentioned earlier. However, the role of NO in the context of antioxidant status is much more complex, so that this effect of grape polyphenols should probably receive more attention before it can be interpreted fully in terms of mechanism(s) involved. Furthermore, recently in a coculture simulation of the human blood-brain barrier, another grape polyphenol, proanthocyanidin, was associated with significant inhibition of monocyte infiltration and proinflammatory cytokine secretion in HIV-associated neuroinflammation [64]. Such inhibition of neuroinflammation is associated with a better prognosis in terms of HIV-related neurodegeneration and dementia, further confirming the neuroprotective potential of grape-derived antioxidants. Taken together, these studies suggest that the neuroprotective effects of grape polyphenols involve both antioxidant and anti-inflammatory mechanisms, with the latter including not only modulated cytokine signalling, but also modulation of both motility and functional capacity of leukocytes, as previously illustrated by our group [64, 65].

**Figure 1:**
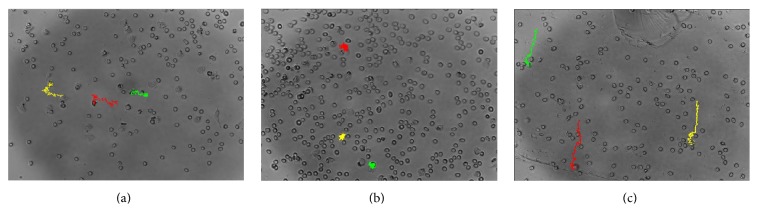
Typical neutrophil chemotaxis pathways for (a) a young participant (<25 yr), (b) an aged participant (>65 yr), and (c) an aged participant after acute* in vitro* treatment with grape-deed derived proanthocyanidin. The Olympus Cell® system IX-81 inverted fluorescent microscope system with an F-view cooled CCD camera (Soft Imaging Systems) at 20x magnification was used to capture these images, which was analysed using Image J (Java software).

One may argue that both these results may be the result of decreased cell activation, perhaps as a result of the known altered cytokine environment. However, an age-associated lack of neutrophil chemokinetic accuracy in response to the chemotaxin fMLP has been reported [49], which suggests that the mechanism is probably related to age-induced compromise of specific cellular mechanisms, rather than activation. In addition, in an ongoing study by our group, we have been able to illustrate by using Dunn chamber chemokinetic assays that grape polyphenols (specifically proanthocyanidin) are able to correct this age-associated anomaly (unpublished data). From Figure 1, which depicts typical digital images obtained for the path of individual neutrophils, it is clear that a more purposeful, directionally accurate movement was achieved in proanthocyanidin-treated neutrophils. This will ensure a more optimal inflammatory response (i.e., the response will be effective, result in relatively insignificant secondary damage, and be resolved in the minimum amount of time). In contrast the rather “aimless wander” of untreated neutrophils from aged individuals will result in not only ineffective immune cell infiltration to sites where they are required, but also relatively more secondary tissue damage and thus prolonged and exacerbated inflammation.

Turning attention to inflammation and oxidative stress again, the most probable targets for therapeutic intervention in the context of ageing have been described in Section 4. Since the ageing literature is relatively lacking in terms of papers on polyphenol intervention, we have tabulated effects of grape polyphenols reported for these identified targets in Table 1, citing relevant data that was mostly obtained in models other than ageing. The aim with this table was not to present all studies on grape-derived products; rather, it is an attempt to show the many models, species, and disease systems in which beneficial effects on oxidative stress and inflammatory status have consistently been reported. Also, although we have included doses used for* in vivo* studies for general comparison and again to illustrate the large variations in doses, these doses are product/extract specific and cannot be extrapolated across board.

From Table 1, which is by no means a complete list of all studies reporting on grape-derived substances, grape-derived products are undeniably beneficial in limiting the magnitude of the inflammatory response as well as to increase antioxidant activity and seem to have multiple targets.

Of course, when considering potential antiageing modalities, it is also of interest to include evaluation of changes in quality of life. Since ageing is associated with natural muscle wasting or sarcopenia [83], it is important to note that, in this context, supplementation with grape polyphenols (50 mg/kg/day) for 4 weeks mitigated skeletal muscle atrophy in a mouse model of chronic inflammation [84]. This was achieved via modulation of two distinct pathways: one directly linked to inflammation (decreased NF-*κ*B activation) and the other due to antioxidant function (limited ROS-associated mitochondrial damage and caspase-3 and -9 activation). Since caspase-3 activation is also a known proapoptotic signal [85], reduced activation and thus apoptosis may result in fewer mitotic cycles. This, in the context of the telomere hypothesis, may point to deceleration of ageing by the polyphenols. Very recently, grape proanthocyanidin treatment in rats was reported to have an antiapoptotic effect which reduced damage after ischemia/reperfusion of the liver [86], which further substantiates this theory.

Interestingly, a study in mice fed a high-fat diet indicated that grape polyphenols may modify gut microbial community structure to result in lower intestinal and systemic inflammation [87]. This extraordinary result serves to remind us of the potential complexity of plant medicines and the requirement for comprehensive investigation of mechanisms of actions and interactions of any potential product* via* the traditional clinical trial process followed for new pharmaceutical drugs.

## 6. Conclusion

Ageing and accelerated ageing are not new concepts, but rather the norm in modern society. With a population that is growing relatively older due to advances in medicine, we are however pressed for answers on how to alleviate the symptoms or slow the progression of this inevitable phenomenon. From the literature consulted, no negative effects of grape-derived products became evident, while beneficial effects in the context of oxidative stress and inflammation were consistently reported in the context of numerous cellular targets. Huge variation in product content and prescribed dosage complicates interpretation of the fast growing body of literature on this topic. A recommendation for future studies is the inclusion of more parameters per study, so that a more comprehensive interpretation of specific mechanisms becomes possible. Measurement of only basic indicators of either antioxidant status or inflammatory status, while providing proof of efficacy of the product, does not contribute much information on its mechanism of action. Despite this shortcoming, the literature clearly indicates that grape-derived products are undeniably a therapeutic force to be reckoned in the combat of ageing and age-associated conditions.

## Figures and Tables

**Table 1:** Representative reports on antioxidant and anti-inflammatory effects of grape-derived crude extracts and purified products.

Model	Treatment	Outcomes: inflammation	Outcomes: oxidative status	References
*In vitro:*				
Glucose and LPS-induced inflammation in HUVEC cells	Red grape polyphenols	↓IL-6, IL-8, and NF-*κ*B at protein and mRNA levels ↓PECAM and ICAM-1 levels	↓ROS formation in dose-dependent manner	[66]
Primary human chondrocytes challenged with *E. coli* LPS (arthritis model)	Grape extract containing resveratrol, hopeaphenol, and viniferin	↓PGE_2_ production	↑scavenging of DPPH radicals	[67]
Osteoblast-like cells (MC3T3-Ei), treated with TGF-*β* to induce VEGF synthesis	Resveratrol	↓VEGF and VEGF mRNA, but no effect on p38 or SAPK/JNK, suggesting SIRT1 activation	n.a.	[68]
Yeast models of sirtuin activation (*c. elegans*, *D. melanogaster)*	Resveratrol	↑sirtuin (SIRT1) activation	n.a.	[69, 70]
Human adipose derived stem cells (hASCs)	Red grape (muscarine) grape seed oil, in comparison to rice bran and olive oils	↓adipogenetic factors (PPAR*γ* and aP2) ↓IL-6 and IL-8 response to LPS ↓proinflammatory gene expression in adipocytes	Shown to be source of *γ*-tocopherol	[71]
High-glucose induced oxidative stress in porcine proximal tubule cells (LLC-PK_1_)	Grape seed polyphenols	↓NF-*κ*B pathway	↓intracellular ROS	[72]
*In vivo* animal:				
Rats exposed to localised bowel irradiation	Grape polyphenols OR pure quercetin 3-*O*-*β*-glucoside (10 mg/mL, 7.14 mL/kg body mass) by oral gavage for 5 days prior to irradiation	↓MPO activity ↓CINC-1 levels	↓SOD activity No change in glutathione peroxidase (GSHPx) activity No change in plasma malondialdehyde (MDA) concentration	[73]
Rats subjected to *E. coli*-induced septic shock	75 and 200 mg/kg/day grape seed procyanidin, by ip. injection for 15 days pre-*E. coli* challenge	↓IL-6 gene expression	↓NO in liver, spleen, plasma, and RBCs ↓iNos gene expression ↓GSSG: total glutathione ratio	[74]
Rat model of osteoarthritis	500 mg/kg body mass of grape extract daily for 28 days	Prevented joint deterioration	n.a.	[67]
Rat model of skeletal muscle contusion injury	Acute OR 2-week supplementation, proanthocyanidins	↓proinflammatory cytokine signalling (TNF-*α*; IL-6) ↓neutrophil migration capacity Earlier macrophage switch from pro- to anti-inflammatory phenotype	↑plasma and skeletal muscle ORAC	[50, 75]
Rat model of ageing	Drinking water supplemented with 15 g/L grape powder for 3 weeks	↓age-associated increase in corticosterone	↓plasma 8-isoprostane	[76]
Rat model of obesity	Grape procyanidin B2	↓IL-1*β* and NLRP3 levels in pancreas	n.a.	[77]
Middle-aged mice on high-calorie diet	Diet supplemented with 0.04% resveratrol	↓IGF-1	↑AMPK and PGC-1*α* activity ↑mitochondrial number	[78]
Mouse model of pulmonary fibrosis	7-day oral resveratrol (50 mg/kg/day) OR quercetin OR dihydroquercetin (both 10 mg/kg/day)	↓neutrophil infiltration into lung tissue ↓inflammatory cells in bronchoalveolar lavage fluid ↓COX-2 ↓NF-*κ*Bp65 translocation	↓iNOS ↓oxidative lung damage (↓nitrotyrosine and poly-ADP-ribose polymerase levels)	[79]
Rabbit model of acute (*E. coli*) inflammatory arthritis	500 mg/kg body mass of extract acutely prior to *E. coli* challenge	↓PGE_2_ production	n.a.	[67]
*In vivo* human:				
Nondiabetic haemodialysis patients	Grape powder (500 mg polyphenols/day) for 5 weeks	Prevented increase in plasma CRP levels	↑glutathione peroxidase activity	[80]
Humans at risk for metabolic syndrome, aged 30–65	16 weeks of 20 g wine grape pomace powder (822 mg polyphenols) per day	n.a.	↑*γ*- and *δ*-tocopherol	[81]
Hypertensive, T2DM males, aged ≈55–65	8 mg grape extract daily for 1 year	↓alkaline phosphatase ↓TNF-*α*, IL-1*β*, IL-6, and CCL3 levels ↑transcriptional repressor LRRFIP-1 in PBMCs Modulation of expression of group of miRNAs known to regulate inflammatory response	n.a.	[82]

ADP, adenosine diphosphate; AMPK, adenosine monophosphate-activated protein kinase; CCL3, chemokine(C-C motif) ligand 3; CINC-1, cytokine-induced neutrophil chemoattractant-1; COX-2, cyclooxygenase-2; DPPH, 2,2-diphenyl-1-picrylhydrazyl; HUVEC, human umbilical vein endothelial cells; iNOS, inducible nitric oxide; ICAM-1, intercellular adhesion molecule-1; IGF-1, insulin growth factor-1; IL, interleukin; JNK, c-Jun N-terminal kinase; LPS, lipopolysaccharides; LRRFIP-1, leucine-rich repeat in flii-interacting protein-1; NO, nitric oxide; NF-*κ*B, nuclear factor-kappa beta; ORAC, oxygen radical absorbance capacity; PECAM, platelet endothelial adhesion molecule; PGC-1*α*, peroxisome proliferator activated receptor gamma coactivator 1-alpha; PGE_2_, prostaglandin E_2_;  ROS, reactive oxygen species; SAPK, stress activated protein kinase; VEGF, vascular endothelial growth factor.
